# Supplementary material for: All-Atom Molecular Dynamics Simulations Indicated the Involvement of a Conserved Polar Signaling Channel in the Activation Mechanism of the Type I Cannabinoid Receptor
Source: Int J Mol Sci. 2023 Feb 20;24(4):4232. doi: 10.3390/ijms24044232 (PMC9963961; doi:10.3390/ijms24044232)
Supplement: Supplementary file 1 [file ijms-24-04232-s001.zip › ijms-2208894-supplementary.pdf]

Supporting Information for

All-Atom Molecular Dynamics Simulations Indicated the Involvement of a Conserved Polar Signaling Channel in the Activation Mechanism of the Type I Cannabinoid Receptor

Arijit Sarkar[1,2], Argha Mitra[1,2], Attila Borics[1,].*

[1]Laboratory of Chemical Biology, Institute of Biochemistry, Biological Research Centre, Szeged, 62. Temesvári krt., Szeged, Hungary, H-6726.

[2]Theoretical Medicine Doctoral School, Faculty of Medicine, University of Szeged, 97. Tisza L. krt., Szeged, Hungary, H-6722.

***Corresponding Author**

Email: borics.attila@brc.hu, Phone: +36 62 599 600 ext. 430.

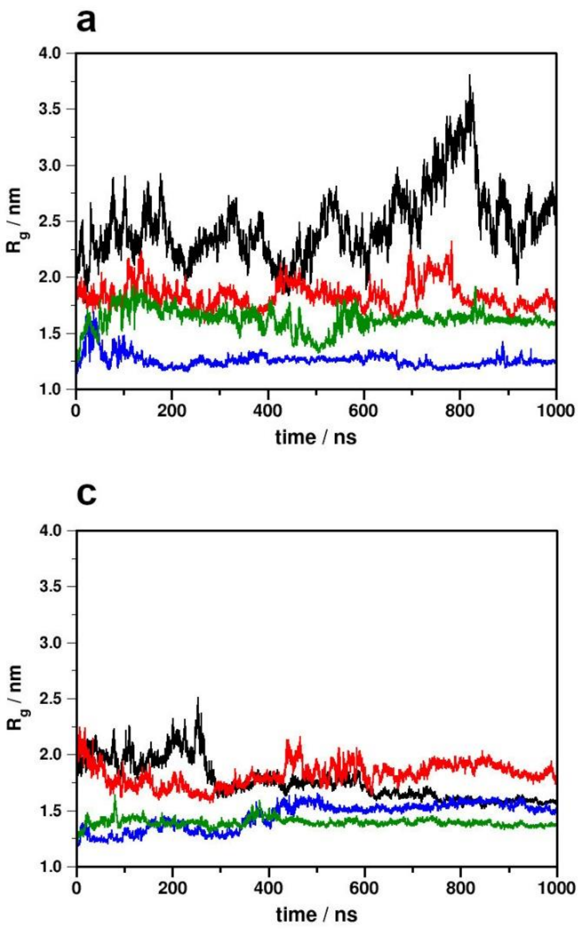

Figure S1. The evolution of radii of gyration of the N- and C-terminal domains of the CB1 receptor during production simulations. (a) active CB1 – G_i protein complex; (b) active CB1 – β-arrestin-2 complex; (c) inactive CB1 – G_i protein complex; (d) inactive CB1 – β-arrestin-2 complex. Black: N-terminal, 1st replica; red: N-terminal, 2nd replica; blue: C-terminal, 1st replica, green: C-terminal, 2nd replica.

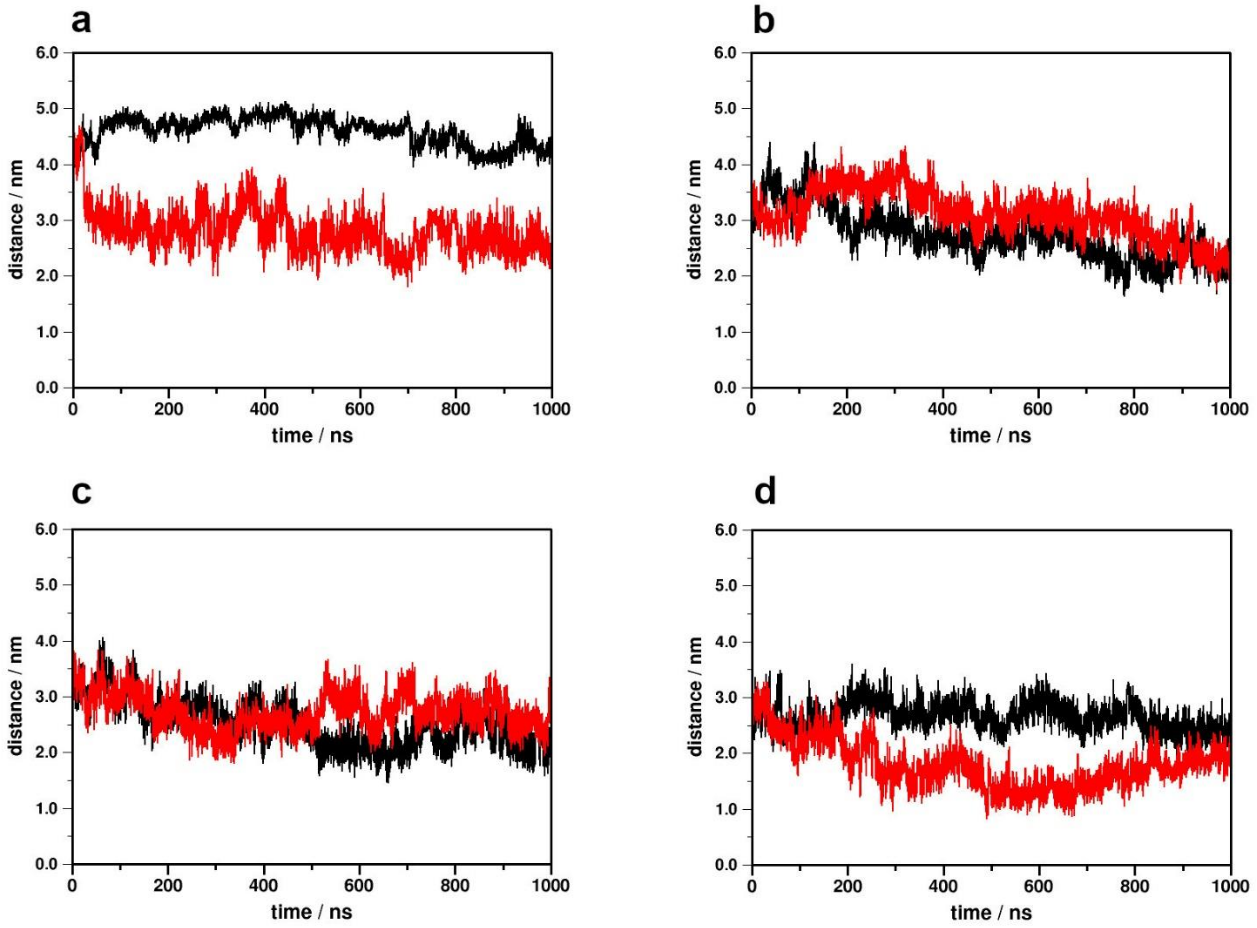

Figure S2. Minimum distance between the N- and C-terminal domains (and their periodic images) of the CB1 receptor during production simulations. (a) active CB1 – G_i protein complex; (b) active CB1 – β-arrestin-2 complex; (c) inactive CB1 – G_i protein complex; (d) inactive CB1 – β-arrestin-2 complex. Black 1st replica; red: 2nd replica.

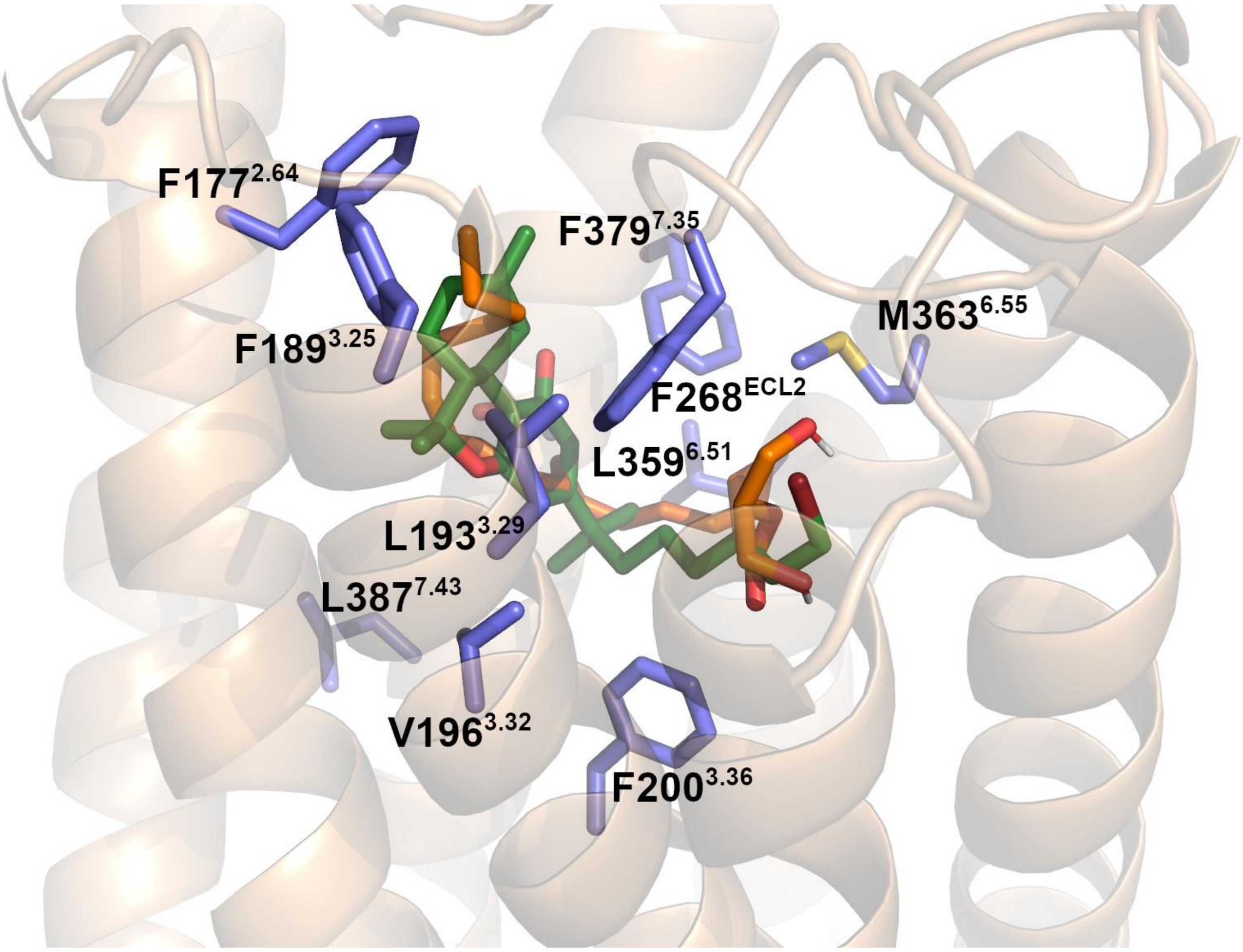

Figure S3. Comparison of the constructed orientation of 2-AG (orange), obtained from blind docking, to the crystallographic structure of AM11542 (green, pdb code: 5XRA) in the orthosteric binding pocket of the CB1 receptor. Amino acid side chains in contact with AM11542 in the experimental structure are shown as slate sticks and labeled.

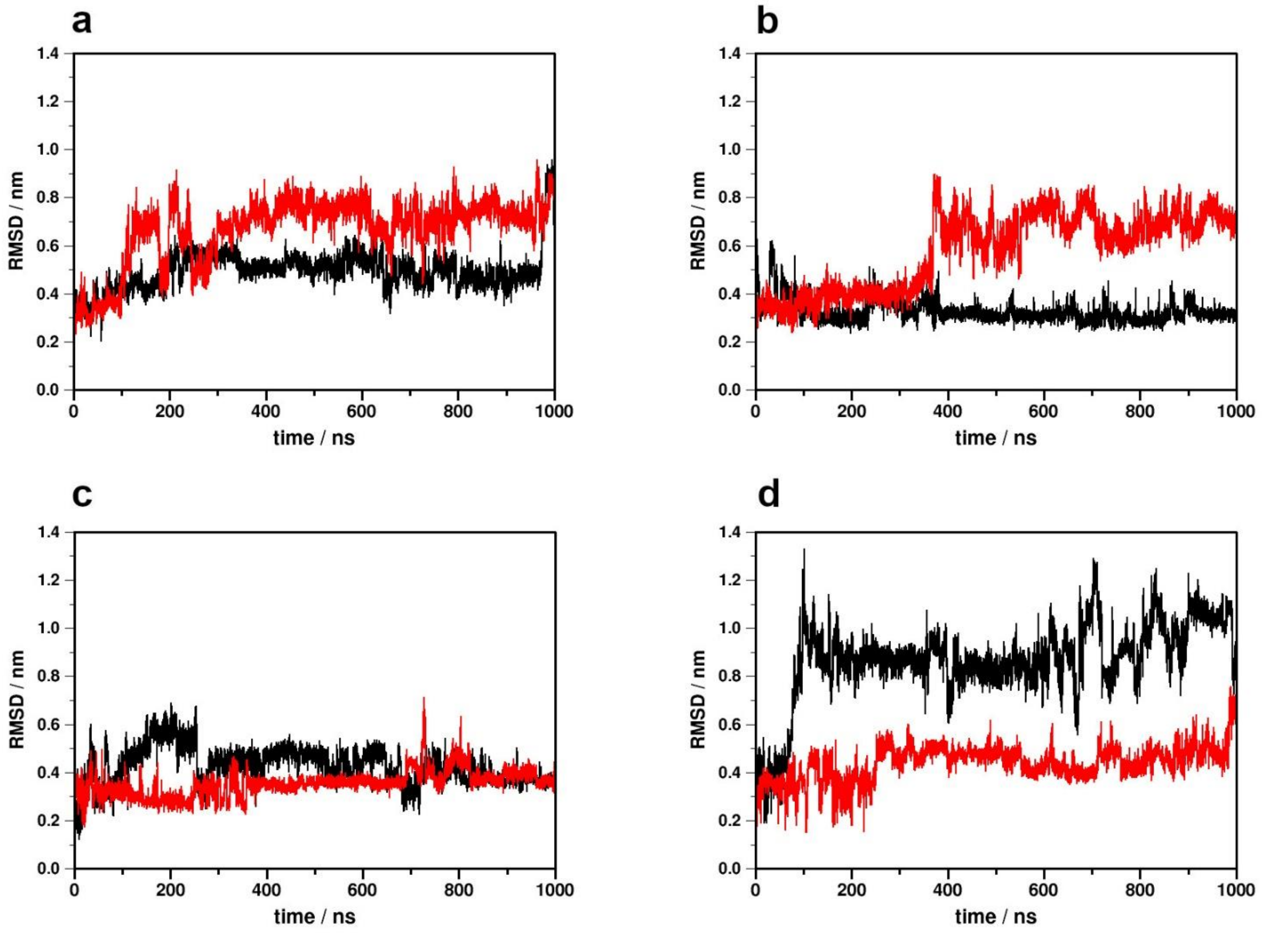

Figure S4. Disposition of 2-AG from its initial conformation and orientation during simulations. (a) active CB1 – G_i protein complex; (b) active CB1 – β-arrestin-2 complex; (c) inactive CB1 – G_i protein complex; (d) inactive CB1 – β-arrestin-2 complex. Black 1st replica; red: 2nd replica.

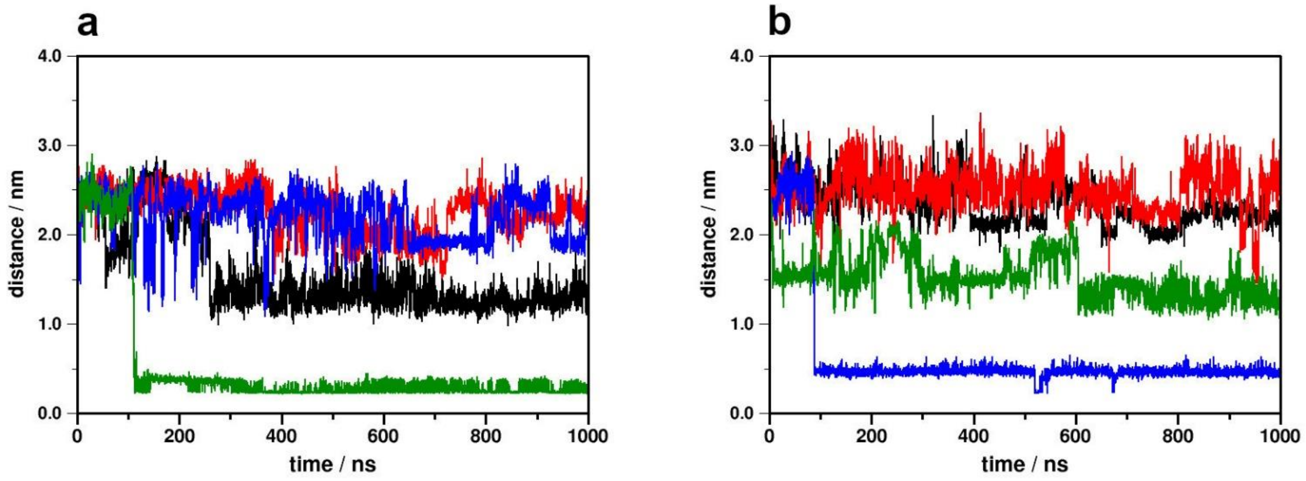

c

	G_i protein complex				β -arrestin-2			
	active state		inactive state		active state		inactive state	
	replica 1	replica 2	replica 1	replica 2	replica 1	replica 2	replica 1	replica 2
Na^+ within 0.4 nm of $D163^{2.50}$	0.0	0.0	0.0	86.6	0.0	0.0	37.8	0.0

Figure S5. Minimum distance between Na^+ ions and $D163^{2.50}$ of the allosteric binding pocket of the CB1 – G_i protein (a) and CB1 - β-arrestin-2 complexes (b). Black: active, 1st replica; red: active 2nd replica; blue: inactive, 1st replica; green: inactive 2nd replica. (c) The frequency of Na^+ present in the allosteric site during simulations.

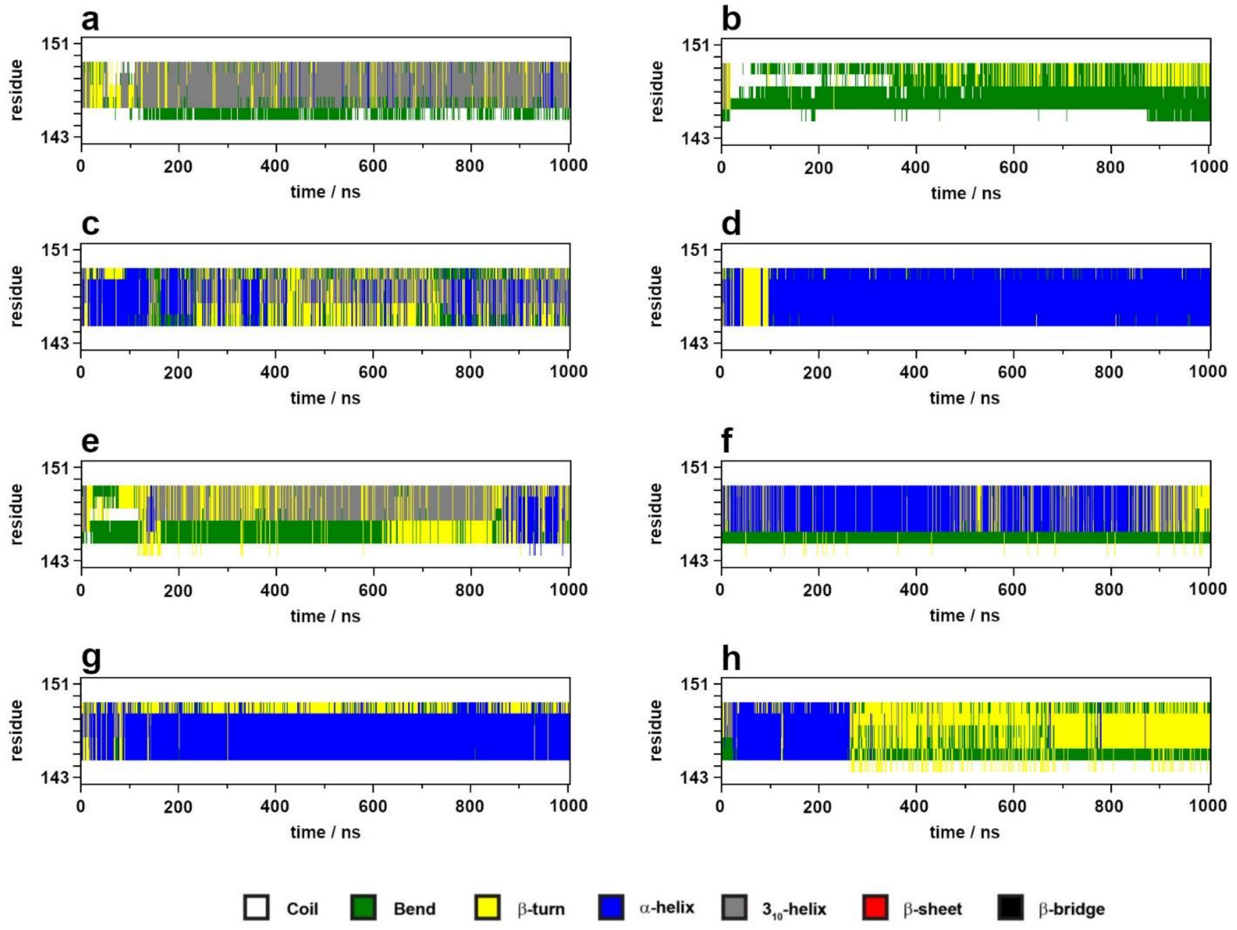

Figure S6. Evolution of the secondary structure of ICL1 during simulations. (a) active CB1 – G_i protein complex, 1st replica; (b) active CB1 – G_i protein complex, 2nd replica; (c) inactive CB1 – G_i protein complex, 1st replica; (d) inactive CB1 – G_i protein complex, 2nd replica; (e) active CB1 – β-arrestin-2 complex, 1st replica; (f) active CB1 – β-arrestin-2 complex, 2nd replica; (g) inactive CB1 – β-arrestin-2 complex, 1st replica; (h) inactive CB1 – β-arrestin-2 complex, 2nd replica.

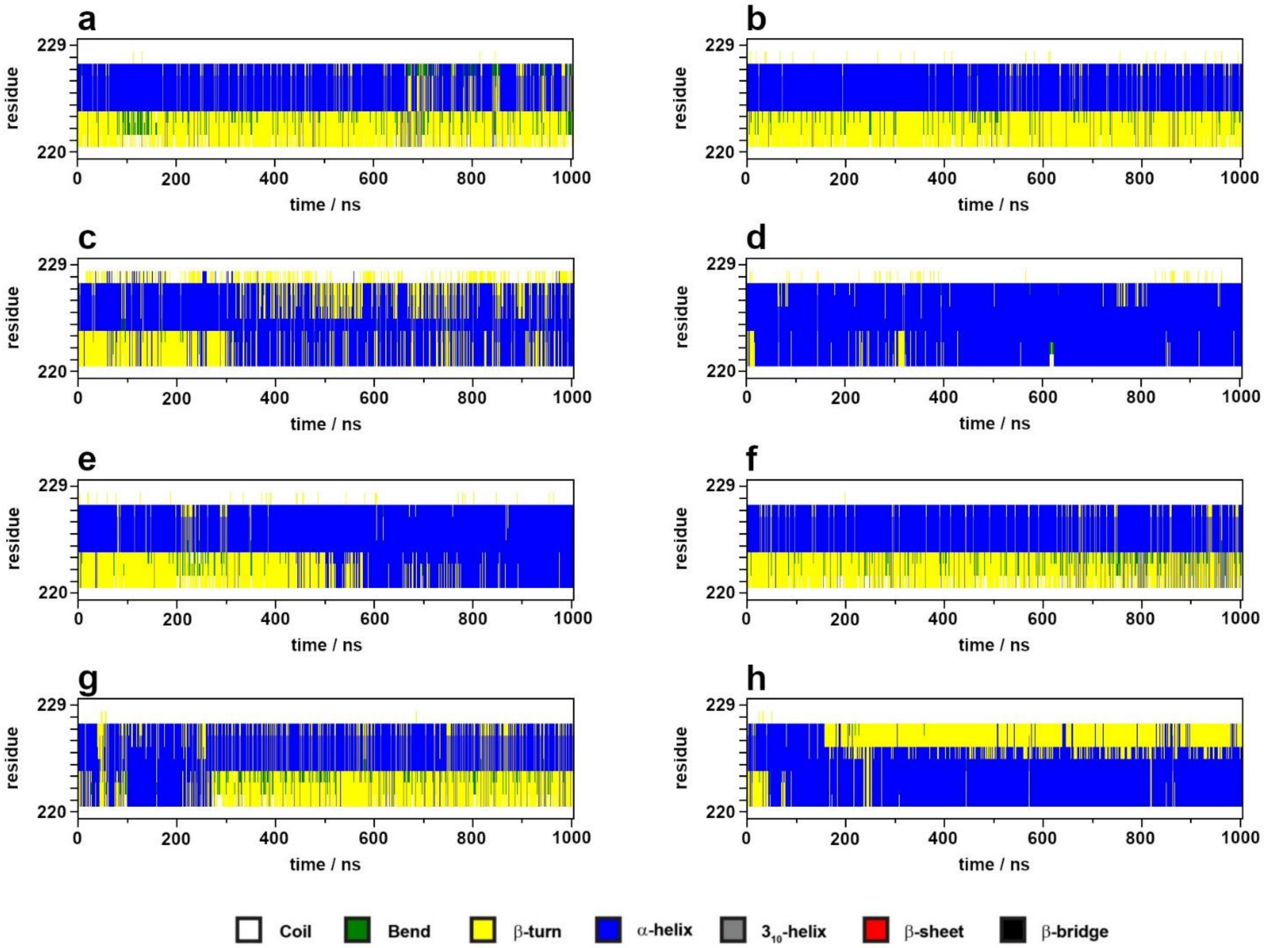

Figure S7. Evolution of the secondary structure of ICL2 during simulations. (a) active CB1 – G_i protein complex, 1st replica; (b) active CB1 – G_i protein complex, 2nd replica; (c) inactive CB1 – G_i protein complex, 1st replica; (d) inactive CB1 – G_i protein complex, 2nd replica; (e) active CB1 – β-arrestin-2 complex, 1st replica; (f) active CB1 – β-arrestin-2 complex, 2nd replica; (g) inactive CB1 – β-arrestin-2 complex, 1st replica; (h) inactive CB1 – β-arrestin-2 complex, 2nd replica.

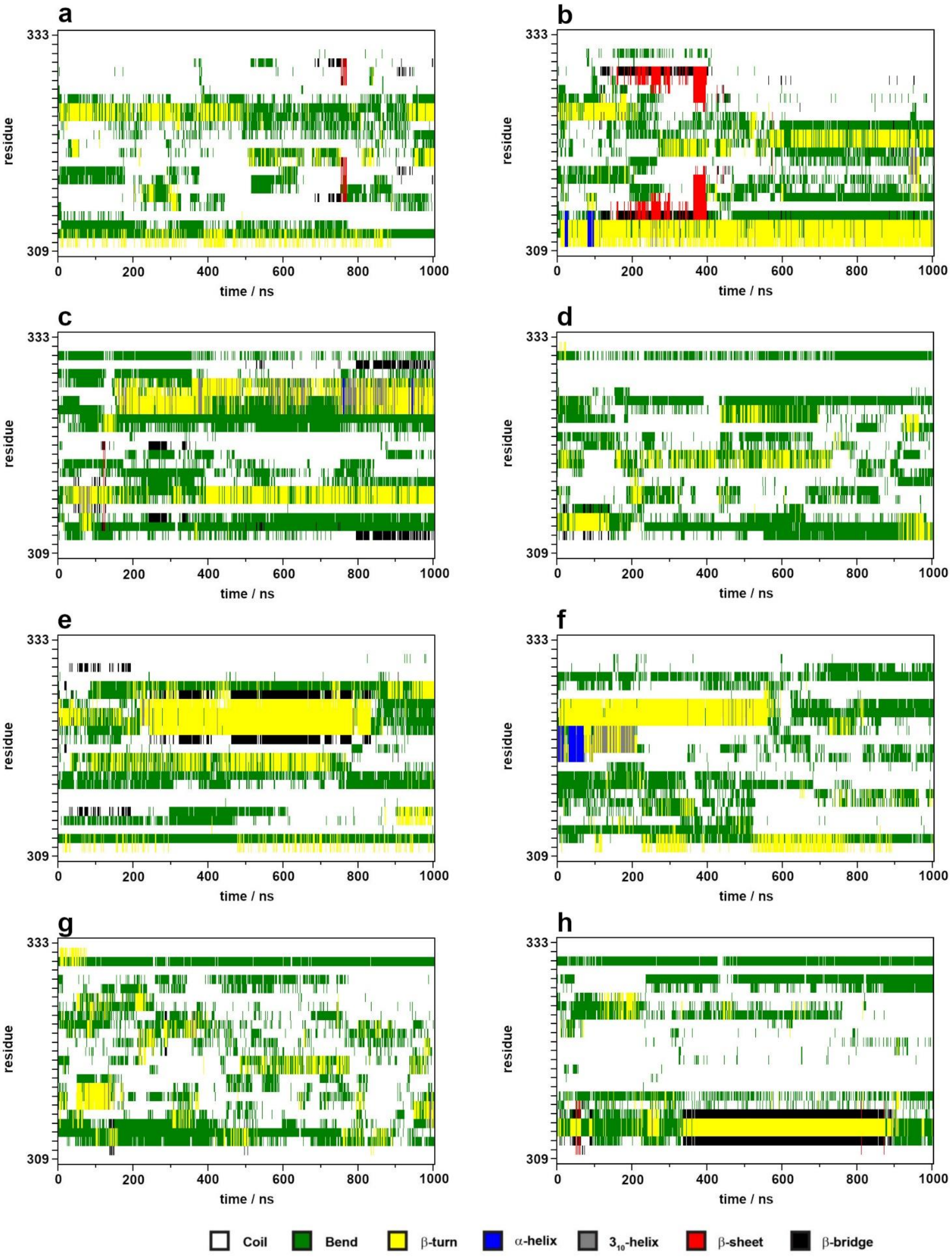

Figure S8. Evolution of the secondary structure of ICL3 during simulations. (a) active CB1 – G_i protein complex, 1st replica; (b) active CB1 – G_i protein complex, 2nd replica; (c) inactive CB1 – G_i protein complex, 1st replica; (d) inactive CB1 – G_i protein complex, 2nd replica; (e) active CB1 – β-arrestin-2 complex, 1st replica; (f) active CB1 – β-arrestin-2 complex, 2nd replica; (g) inactive CB1 – β-arrestin-2 complex, 1st replica; (h) inactive CB1 – β-arrestin-2 complex, 2nd replica.

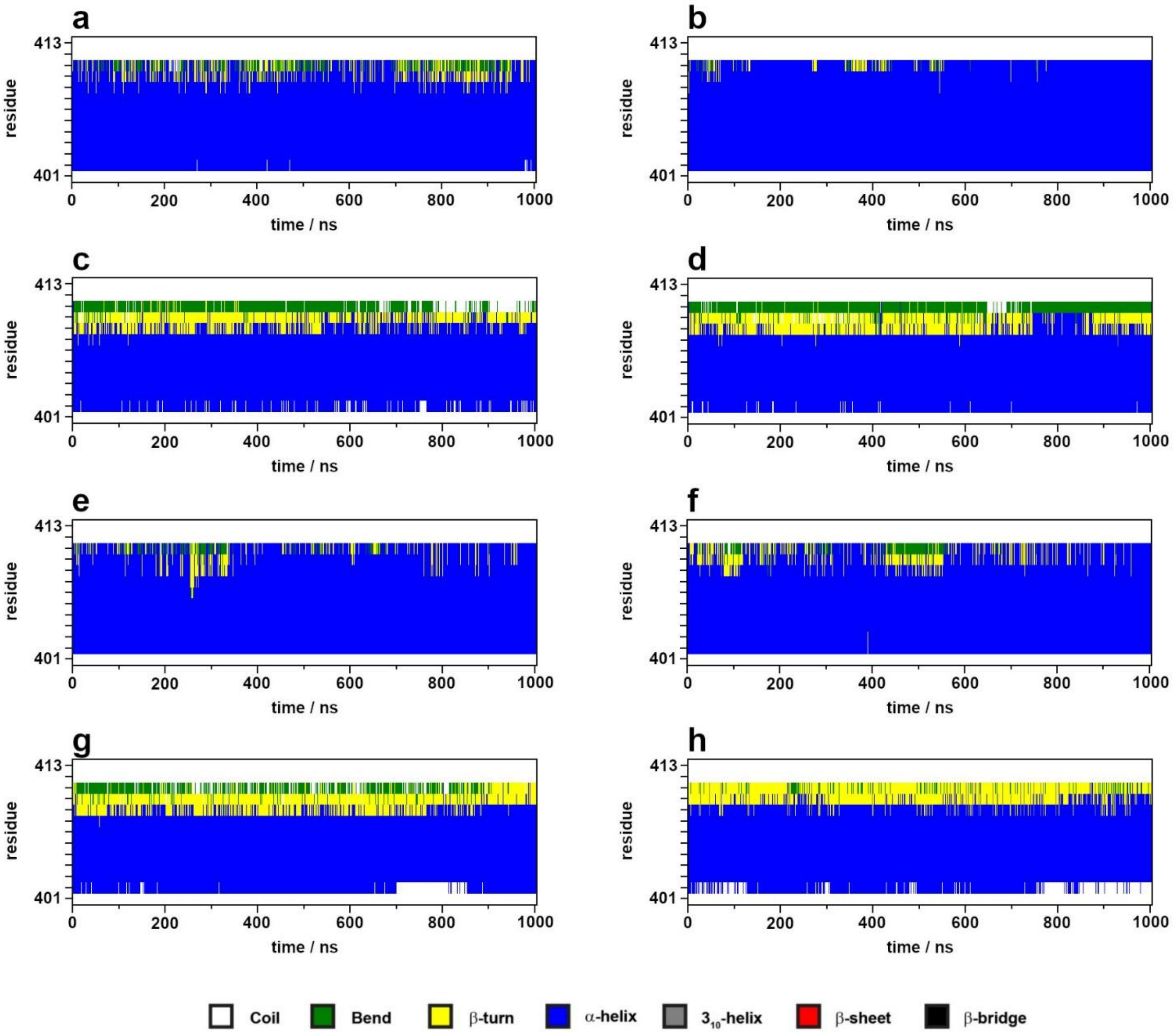

Figure S9. Evolution of the secondary structure of H8 during simulations. (a) active CB1 – G_i protein complex, 1st replica; (b) active CB1 – G_i protein complex, 2nd replica; (c) inactive CB1 – G_i protein complex, 1st replica; (d) inactive CB1 – G_i protein complex, 2nd replica; (e) active CB1 – β-arrestin-2 complex, 1st replica; (f) active CB1 – β-arrestin-2 complex, 2nd replica; (g) inactive CB1 – β-arrestin-2 complex, 1st replica; (h) inactive CB1 – β-arrestin-2 complex, 2nd replica.

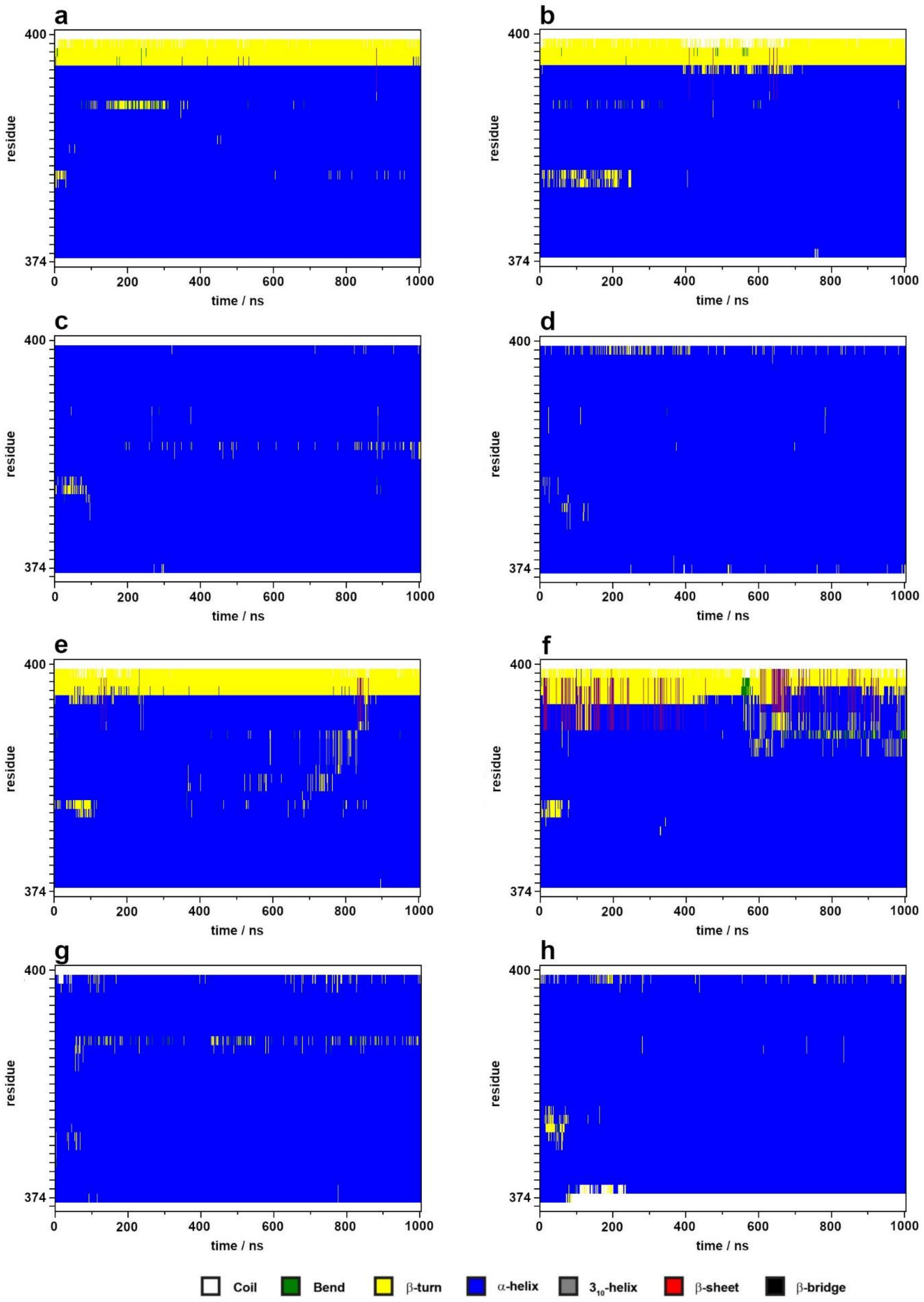

Figure S10. Evolution of the secondary structure of TM7 during simulations. (a) active CB1 – G_i protein complex, 1st replica; (b) active CB1 – G_i protein complex, 2nd replica; (c) inactive CB1 – G_i protein complex, 1st replica; (d) inactive CB1 – G_i protein complex, 2nd replica; (e) active CB1 – β-arrestin-2 complex, 1st replica; (f) active CB1 – β-arrestin-2 complex, 2nd replica; (g) inactive CB1 – β-arrestin-2 complex, 1st replica; (h) inactive CB1 – β-arrestin-2 complex, 2nd replica.

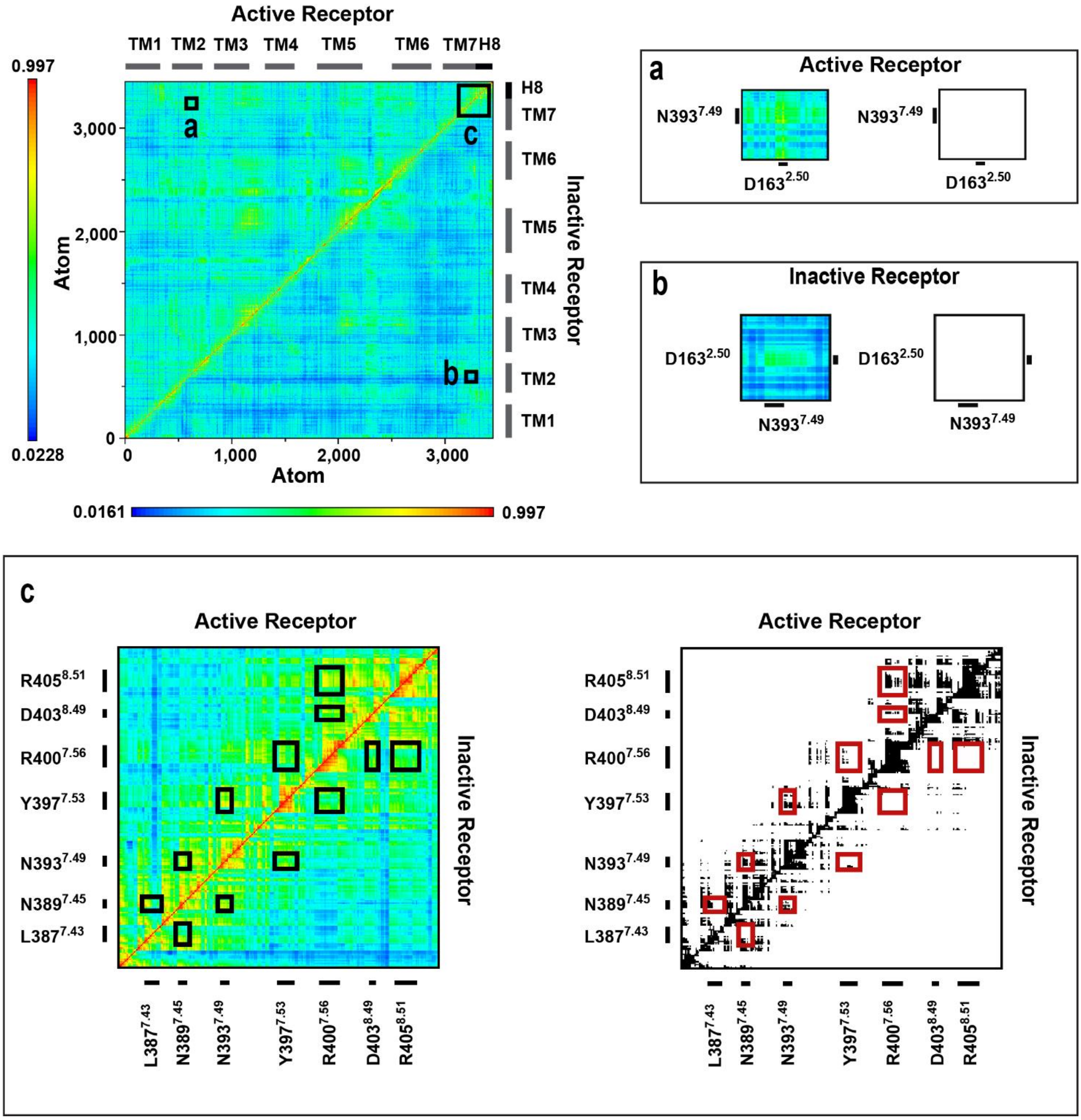

Figure S11. Cross-correlation matrices of the G_i protein-bound CB1 (2nd replica) in the active and inactive states. Panels (a-c) are magnified views of regions of amino acid residues of interest. Black and white panels show correlations above the threshold of 0.63 MI.

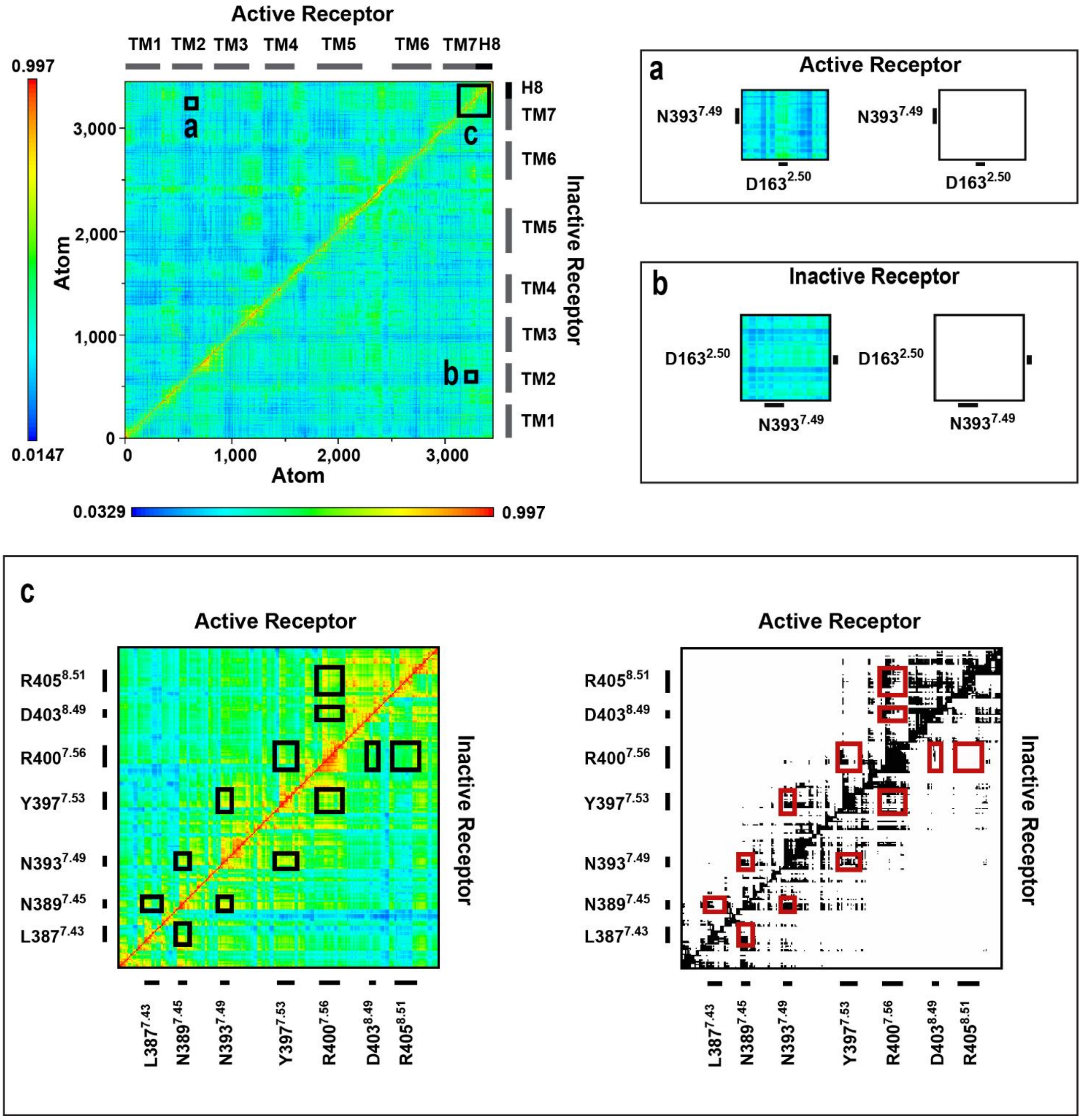

Figure S12. Cross-correlation matrices of the β-arrestin-2-bound CB1 (1st replica) in the active and inactive states. Panels (a-c) are magnified views of regions of amino acid residues of interest. Black and white panels show correlations above the threshold of 0.63 MI.

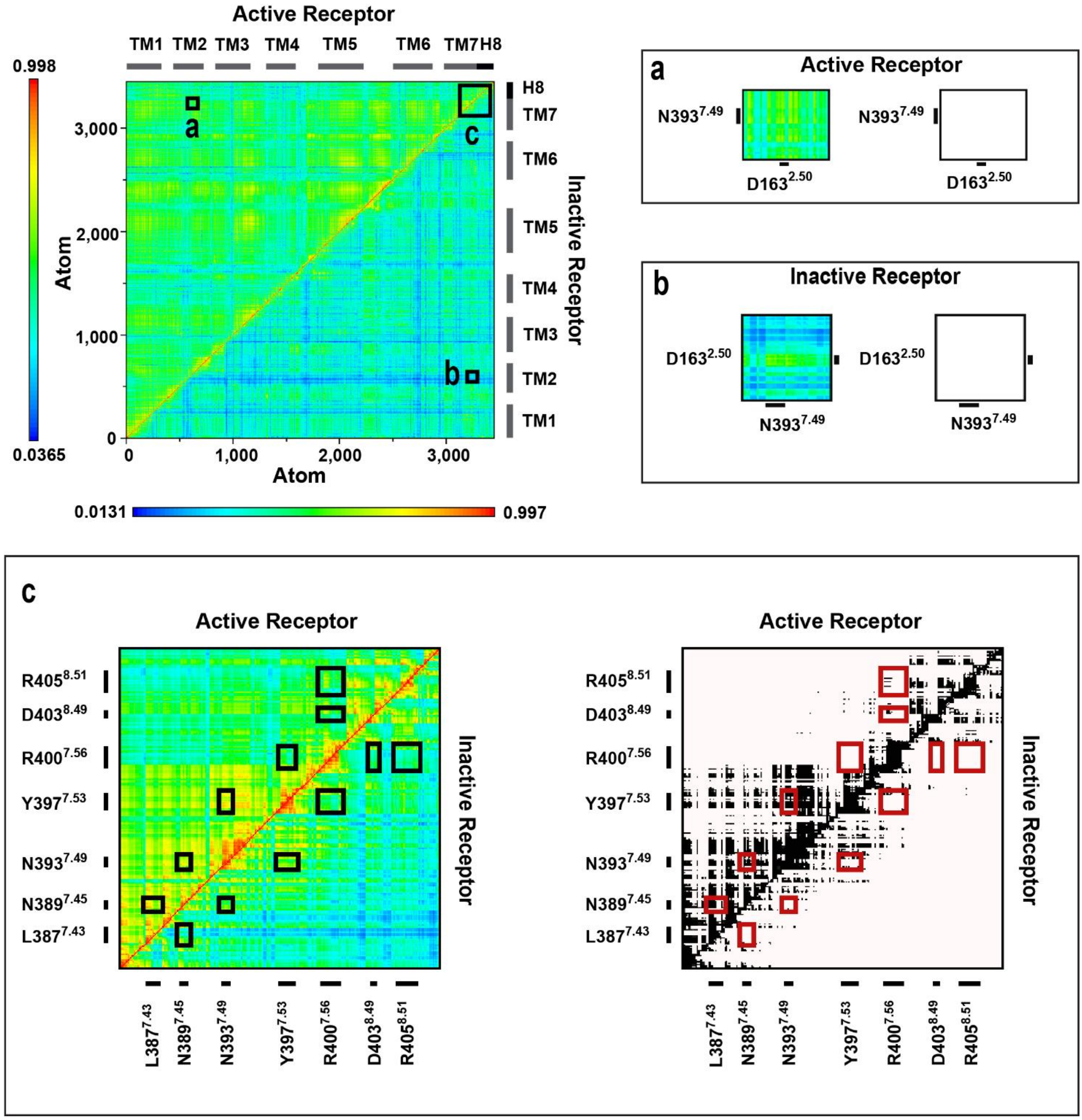

Figure S13. Cross-correlation matrices of the β-arrestin-2-bound CB1 (2nd replica) in the active and inactive states. Panels (a-c) are magnified views of regions of amino acid residues of interest. Black and white panels show correlations above the threshold of 0.63 MI.

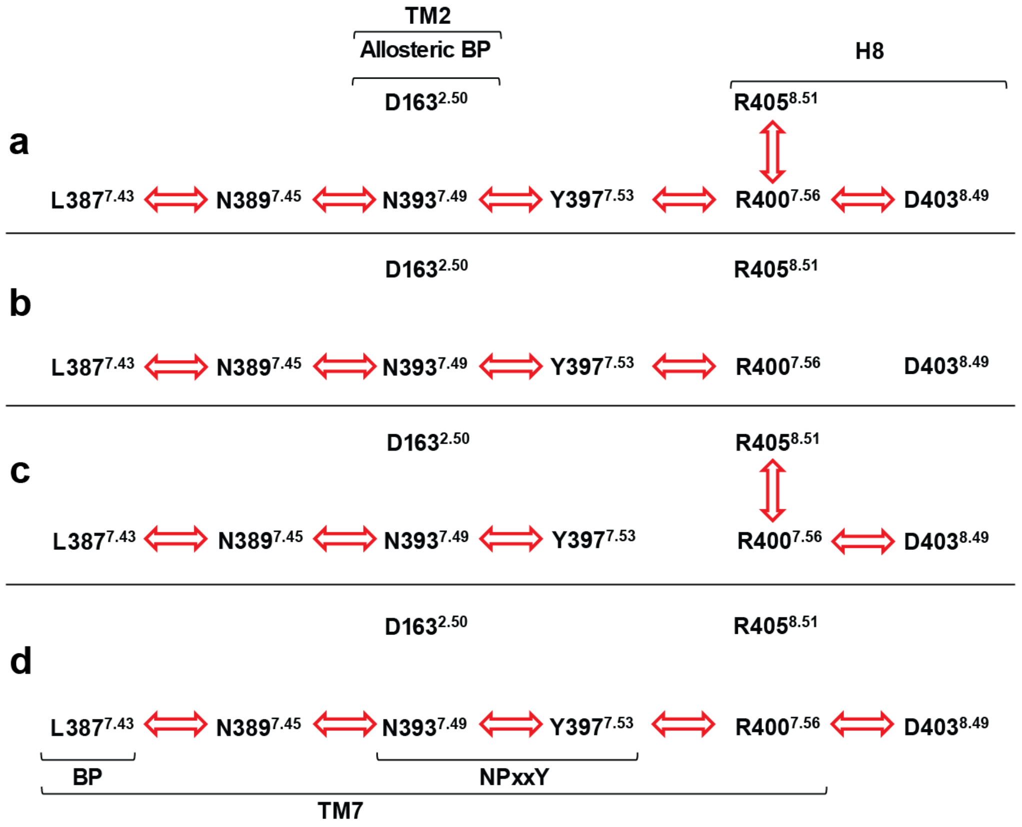

Figure S14. The polar signaling channel of the G_i protein or β-arrestin-2-bound CB1 indicated by cross correlation analysis. (a) active CB1 – G_i protein complex, 2nd replica; (b) inactive CB1 – G_i protein complex, 1st replica; (c) active CB1 – β-arrestin-2 complex, 2nd replica; (d) inactive CB1 – β-arrestin-2 complex, 1st replica. Red arrows indicate correlated motions of the respective amino acids.

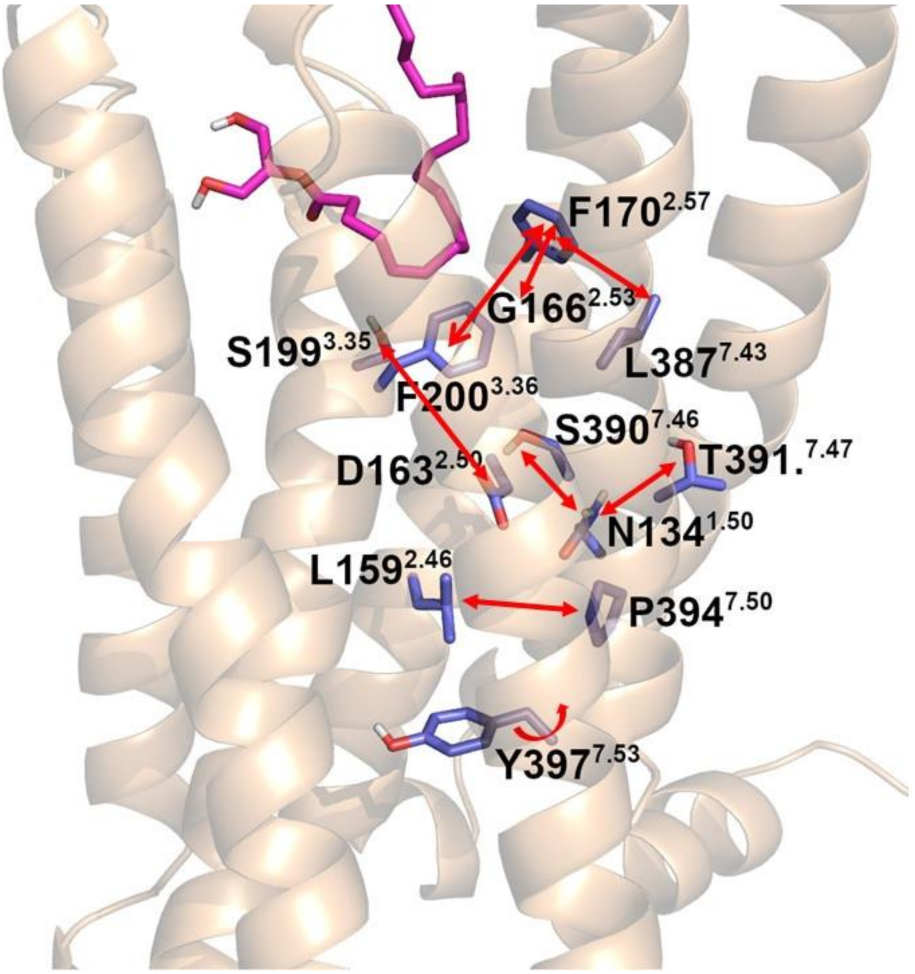

Figure S15. Residues and residue-residue pairs of which side chain conformations and distances were used, respectively, to characterize the two distinct structural states involved in G_i protein and β-arrestin-2 mediated signaling. Straight red arrows indicate characteristic distances. The curved arrow shows the side chain dihedral angle involved in switching between the two active signaling states.

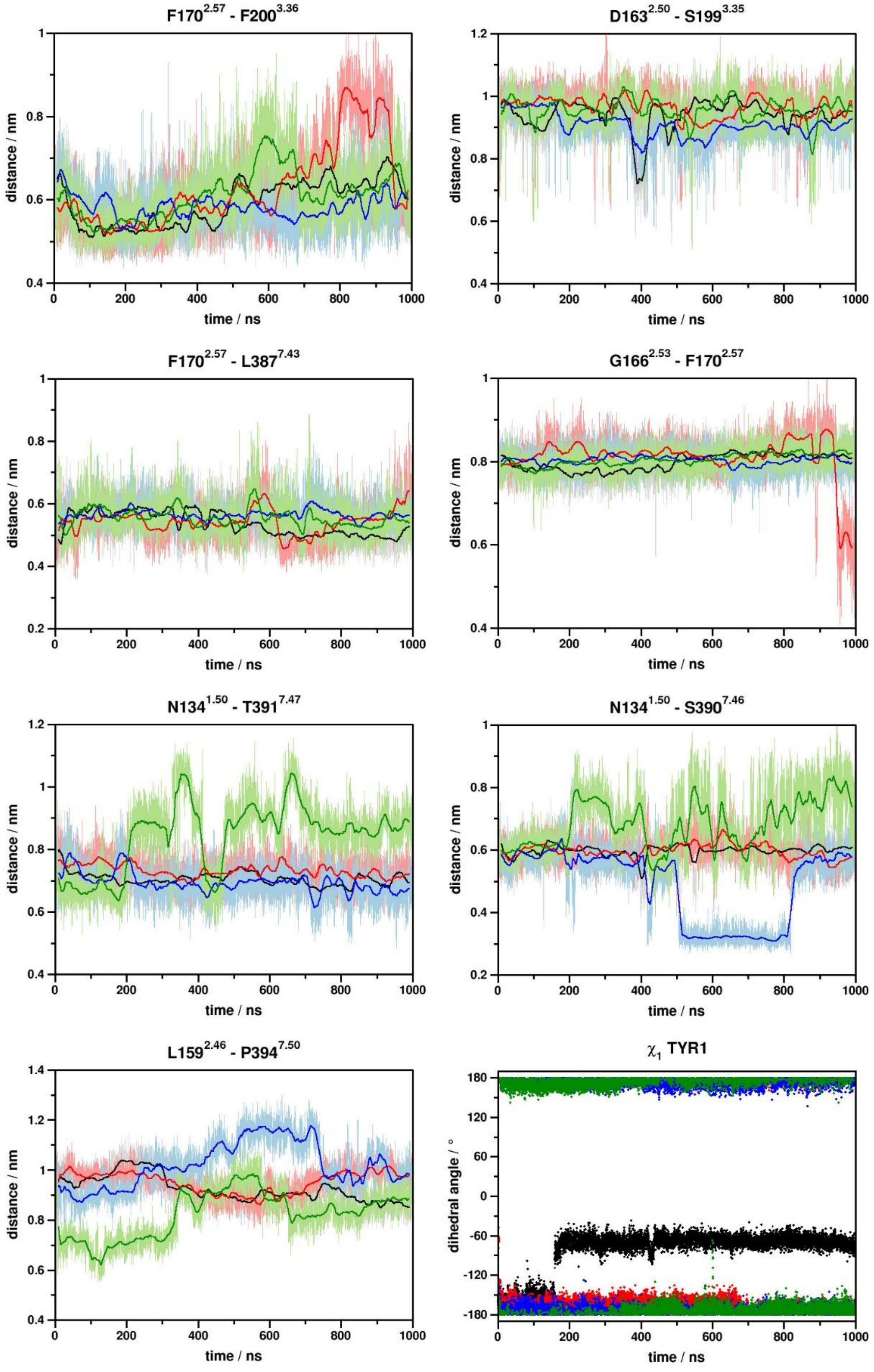

Figure S16. Evolution of specific residue-residue distances and side chain conformations associated with the two distinct structural states involved in G_i protein and β-arrestin-2 mediated signaling. Black: active CB1 – G_i protein complex, 1st replica; red: active CB1 – G_i protein complex, 2nd replica; blue: active CB1 – β-arrestin-2 complex, 1st replica; green: active CB1 – β-arrestin-2 complex, 2nd replica.
